# Understanding Public Health Adaptation to Climate Change: An Explorative Study on the Development of Adaptation Strategies Relating to the Oak Processionary Moth in The Netherlands

**DOI:** 10.3390/ijerph18063080

**Published:** 2021-03-17

**Authors:** Yvette Buist, Marleen Bekker, Lenneke Vaandrager, Maria Koelen

**Affiliations:** Department of Social Sciences, Health and Society, Wageningen University & Research, P.O. Box 8130, Bode 60, 6700 EW Wageningen, The Netherlands; marleen.bekker@wur.nl (M.B.); lenneke.vaandrager@wur.nl (L.V.); koelen@caiway.nl (M.K.)

**Keywords:** public health adaptation, oak processionary moth, actor map, organisation, values

## Abstract

Understanding of public health adaptation (PHA) to climate change and implementation is limited. This study therefore focuses on one specific PHA issue: adaptation to the oak processionary moth (OPM). The aim is to examine the development of OPM adaptation in order to offer a problem description of the complexities involved in OPM adaptation. In this explorative case study, we investigate adaptation strategies based on semi-structured interviews with 26 actors involved in OPM adaptation in The Netherlands. The results indicate that the context of OPM adaptation is relatively complex, given the involvement of many interdependent actors. OPM adaptation was developed with limited knowledge and strategies were based on ad hoc approaches in which there was ambiguity about tasks and expertise. In addition, different actors have different perceptions and values concerning health, sustainability, risks and responsibilities influencing decision-making processes, while also posing a challenge to collaboration and the development of a coordinated approach. The generation of knowledge and its translation into practical strategies calls for interdisciplinary cooperation in knowledge development. PHA adaptation involves more than technical and organisational solutions alone. It also entails the development of a shared problem perception and solution space in which citizens are also engaged.

## 1. Introduction

Climate change is becoming increasingly recognised as a serious, worldwide public health concern [1]. In the coming years, it is expected to lead to increases in the number of allergies, respiratory complaints, heat and UV-related illnesses and deaths, outbreaks of water, food-borne and vector-borne diseases, and psychological complaints [2,3,4,5,6]. Appropriate responses are therefore needed in order to act on the threats to the wide range of factors related to climate change that affect health and well-being [1,7].

To reduce these threats to human health and well-being, numerous appeals have been made for public health adaptation (PHA) [1,8,9,10,11]: the process of adjusting to actual or expected climate change and its health effects [12]. Strategies for PHA range from technical and institutional solutions to collaborative learning, aimed at objectives including strengthening disease surveillance, establishing interdisciplinary, implementing adaptation plans, establishing working groups and co-generating knowledge on adaptation options [13,14,15,16]. Despite the growing attention to PHA, adaptations are being implemented only on a limited scale [1,8,17,18,19]. As argued by Ebi, Hess [20] and by Watts, Adger [21], PHA poses a challenge to health officials, decision-makers and practitioners, as they must cope with multi-faceted causal changes (e.g., the impact of heat waves on the loss of biodiversity, which subsequently increases the incidence of vector-borne diseases) and uncertainty with regard to how the development of PHA strategies could alter future vulnerability to the risks posed by climate change [20,21]. It is therefore necessary to enhance our understanding of the generation and implementation of knowledge relating to PHA [22,23].

The oak processionary moth (OPM: Thaumetopoea processionea) offers a particularly suitable case for studying PHA. The Dutch Knowledge Agenda on Climate Change and Health has identified the OPM as one emerging PHA problem that is exacerbated by climate change [24]. It is expected that the OPM will become a growing public health concern, especially in light of the fact that it is spreading out northwards through Europe [25]. The OPM affects oak trees in open public spaces, as well as on private land and in residential gardens. Direct or airborne contact with the poisonous setae (small, hair-like structures) of the OPM leads to reactions in the skin and mucous membranes. The setae can also cause conjunctivitis, pharyngitis and respiratory distress, and a few people even experience anaphylactic reactions [26,27]. Adaptation strategies can be applied to prevent the health (and other) impact of the OPM. More specifically, OPM adaptation is the process of adjusting to the effects of OPM and preventing their impact on health and other aspects of public interest. The recent increase and spread of OPM might indicate that, in the near future, OPM control might no longer be sufficient to prevent widespread health impact [28,29,30]. It will therefore be important to apply a variety of OPM adaptation strategies.

Strategies for OPM adaptation can be divided into two categories. The first category consists of ecologically focused OPM control measures aimed at reducing OPM. The second category entails human-focused measures aimed at reducing the health impact of the OPM. The focus of the OPM adaptation strategies, the actors responsible and the types of measures are summarised in Table 1. Examples of ecologically focused measures include natural control measures (e.g., increasing biodiversity); the application of organic or chemical agents; the removal of nests and setae; the planning and prioritisation of insecticides or pesticides (including bio-pesticides) [28,31,32,33]. Examples of human-focused measures include informing citizens about OPM, avoiding affected oak trees and indirect contact with setae (e.g., through grass, benches, water), using ointments, and consulting with general practitioners or specialists in case of symptoms. Although some of these adaptation measures have either been substantiated or are evidence-based, it is unclear whether most of them actually work, how they work and/or exactly what their impact is on health of humans and the ecosystem [33,34]. Moreover, as noted by Damestoy, Jactel [31] and, de Klein, Asbreuk [33] effectiveness of OPM adaptation is not based on any single instrument, but rather on a combination of measures, including learning to live with the OPM, given that they are unlikely to be eradicated. In 2019, The Netherlands experienced a major outbreak of OPM [30], which resulted in an increase in people visiting their general practitioners [35]. Tree owners, authorities and citizens had no ready-made solutions at hand to prevent or reduce the health impact of the OPM [28,29]. The outbreak also gave rise to Parliamentary questions calling for a structural national approach and adaptation plan [36].

To date, the literature has focused largely on the ecology and epidemiology of health risks associated with the OPM [28]. To our knowledge, only two studies, both executed in London, have been published on the development of OPM adaptation strategies [28,29]. Tomlinson, Potter [28] identify challenges associated with OPM adaptation, including ambiguities concerning the division of responsibilities, coordination, and debates about significant risks to the health of both trees and humans (2006–2012). Marzano, Ambrose-Oji [29] demonstrate the need for versatility and trade-offs between values (e.g., biodiversity vs. health) in the management of public-health risks and social values in response to the OPM.

To summarise, there is only limited understanding with regard to the generation and implementation of PHA knowledge; the implementation and effects of OPM adaptation are unclear, and difficulties are associated with coordinating an effective response. The objective of this study is therefore to explore and describe the dilemmas and complexities involved in OPM adaptation by mapping actors, networks, activities and challenges involved in OPM adaptation. The aim is to contribute to (a) the development of a conceptual framework to OPM adaptation, (b) to map complexities, to improve understanding of OPM adaptation strategies and (c) make recommendations for further research. This study is based on an in-depth case study set in The Netherlands.

## 2. Methods

This qualitative explorative case study is designed according to a grounded method. This method suits the exploratory character of the study, as it allows key issues to emerge from the data through an inductive approach (Charmaz 1996).

### 2.1. Study Sample

A purposive sampling approach was used, followed by snowball sampling to identify actors who were involved with OPM and to ensure the inclusion of a wide range of actors [37,38]. This approach also supported the objective to map actors, networks, activities and challenges involved in OPM adaptation. First, two municipalities were selected, one located in the East and one located in the South of The Netherlands. These municipalities were chosen because they were heavily affected by the OPM in 2018 and, to an even greater extent, in 2019. Participants were invited by email or by telephone to participate in the study. These actors were either part of the network of the researchers involved in this study or identified through an online search. They were also asked to identify other professionals within their networks who were involved with the OPM. These actors were also invited to participate. We also invited actors involved with the OPM at the national level. In all, 33 actors were invited to participate, and 26 agreed to participate (Table 2). Reasons for non-participation included lack of time and change of professional position. Prior to the interview, participants received information regarding the aims of the interview.

The interviewees worked for municipalities (policy advisor, public greenspace advisor, public space manager, district manager [*n* = 6]), provinces (policymaker, greenspace project manager [*n* = 2]), ministries (policy advisor [*n* = 2]), community health services (physician, medical environmental specialist, social nurse [*n* = 3]), regional site-management organisations (forester, ecologist, team manager [*n* = 4]), a nature association (coordinator, secretary, ecologist [*n* = 3]), a waste operations company (team manager [*n* = 1]), a maintenance contractor (technical manager [*n* = 1]), a sports association (team manager [*n* = 1]) and a general practitioner (*n* = 1). A private tree owner (*n* = 1) and a member of a sports association (*n* = 1) were also included.

### 2.2. Data Collection

The interviews were guided by a semi-structured interview format, which helped the interviewer to think about OPM adaptation topics and enhanced the interviewer’s ability to concentrate while listening during the interviews [39]. The following topics were raised during the interview: the organisation of OPM adaptation, OPM actor networks, coordination and responsibilities, and the development of OPM adaptation strategies. To remain open to the concerns of the interviewees, the interviewer constantly provided them with opportunities to introduce their own themes, concerns and ideas about OPM adaptation [39,40]. Initially, the first author conducted two pilot interviews in order to test the clarity, relevance and sequence of the questions. Based on the pilot interviews, slight adjustments were made to the structure and sequence of the questions. The interviews, including the introduction, lasted between 20 min and 80 min. Interviews were conducted until no new themes emerged, and the researchers agreed that the point of data saturation had been reached [41].

### 2.3. Data Analysis

The interviews were transcribed verbatim and analysed in detail. The data were analysed according to a grounded approach, which provides systematic procedures for the analysis of rich data [42]. In this process, the authors created optimal space for themes and topics raised by the interviewees. A two-step coding process was applied: (1) open coding and (2) focused coding [43]. The first step started with a thorough reading of the interviews and subsequent coding of data with the following questions in mind: ‘What category does this event/situation reflect?’ ‘What is actually happening in the data?’ ‘What is the main concern being raised?’ ‘How can the concerns be explained?’ [40]. In the second step, the codes were sorted into significant themes [43]. The grounded approach enabled the researchers to explicate the collective narrative derived from the interviews, thereby developing a conceptual overview that revealed patterns of action and interaction between and amongst actors [43,44]. The MAXQDA 20.0 software program was used to code the transcripts. Throughout the process, findings were discussed amongst the researchers.

## 3. Results

The inductive approach yielded a rich description of the context within which OPM adaptation takes place. To set the scene, this first sub-section positions actors relevant to OPM adaptation in The Netherlands and describes OPM adaptation networks. The overall results suggest that a distinction can be made between technical, organisational and normative aspects of OPM adaptation and issues related to OPM adaptation. For this reason, the results section continues with a description of the technical issues within which the practice of OPM adaptation is discussed. We then address the organisational aspect of OPM adaptation, which entails preparation; the division of tasks and responsibilities and collaboration within OPM networks; and resources for OPM adaptation. This is followed by a discussion of the normative aspect of OPM adaptation. We identify the underlying value orientations that influence decision-making with regard to OPM adaptation.

### 3.1. Positioning of Actors Relevant to OPM Adaptation in The Netherlands

#### 3.1.1. Actors: Who Is Involved

**Figure 1:**
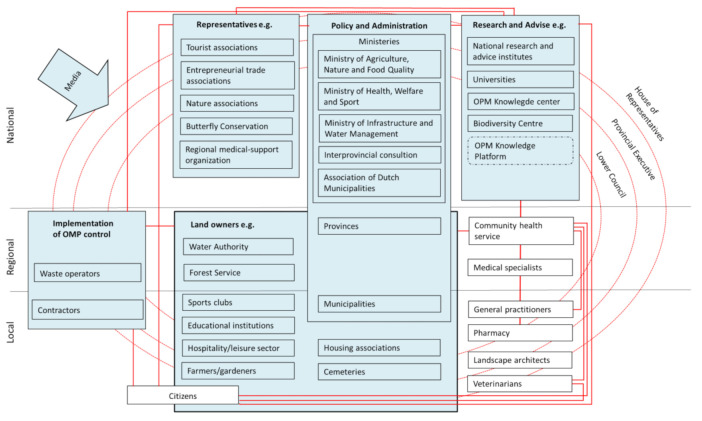
Heuristic representation of actors involved with the OPM.

Actors at the national, regional and local levels are concerned with the problem of the OPM. An overview of the actors involved with OPM adaptation is presented in Figure 1. This map is only an indication and not a complete representation of the relationships that exist between actors in practice. Instead, it is a heuristic illustration of the complexity of the actors. To begin, there is considerable variation amongst landowners, including public tree owners with multiple hectares of trees (e.g., provinces and municipalities) and private tree owners with only one tree (e.g., sports clubs and citizens). All of these owners have the same responsibility to ensure safety around their trees. Provinces and municipalities have a dual role: they are responsible for the land that they own, and they are involved with OPM policy and administration. Citizens can also be concerned with OPM in two ways: citizens owning land with oaks can be affected, as can citizens who do not own private land, but who live or recreate in areas with oaks with OPM. Other landowners can be affected as well, including water authorities; forest services; educational institutions; the hospitality and leisure sectors; farmers and gardeners; housing associations; and cemeteries.

In addition to provinces and municipalities, the Ministry of Agriculture, Nature and Food Quality, the Ministry of Health, Welfare and Sport, the Ministry of Infrastructure and Water Management, the Interprovincial Consultation and the Association of Dutch Municipalities are involved in policy and administration relating to OPM adaptation. Policy and administrative actors are supported by input from research and advisory actors, including national research institutes, universities and the OPM knowledge centre. Representatives of tourist associations, entrepreneurial trade associations, nature associations (including Butterfly Conservation) and community health services can also play a role in informing policy and administration actors, in addition to advocating the rights of employees and tourists. Waste operators and contractors are involved in the implementation of OPM adaptation. Health professionals (e.g., community health services, medical specialists, general practitioners, pharmacists and veterinarians) are concerned with the health effects of the OPM. Landscape architects, hired by public and private landowners, are concerned with the design (and re-design) of outdoor spaces, in which they might need to take OPM into account. The media inform citizens and highlight notable issues concerning the OPM. Moreover, the House of Representatives of The Netherlands, the Provincial Executive and the Lower Council can enact regulations that influence OPM adaptation. The development of PHA strategies for OPM adaptation thus involve the policies of different sectors and actors with various occupational backgrounds.

#### 3.1.2. Collaboration of Actors in Networks

Some of the individual actors interviewed were also collaborating in networks. The networks and connections between the various actors are represented by the red lines in Figure 1. In this section, we elaborate in several examples of OPM adaptation networks. At the national level, there is the OPM Knowledge Centre, which was established in 2012, and in which researchers study the OPM and inform actors of new insights, with the objective of reducing or preventing the ecological and socio-economic effects of the OPM. Another national-level example is the OPM Knowledge Platform OPM, was founded by of the Ministry of Agriculture, Nature and Food Quality in the summer of 2019, in response to Parliamentary questions. In this network, various research, advisory and consultancy actors collaborate to prevent OPM health risks by communicating the latest insights about OPM adaptation. Several working groups have been established within the Knowledge Platform. The Platform collects and shares knowledge and information about liability, OPM waste, control and management, health, OPM knowledge gaps, monitoring and evaluation. The OPM Knowledge Centre is also represented in the OPM Knowledge Platform.

At the regional and local levels, most of the OPM networks have been established since 2019 and, as noted by the interviewees, many of these networks are in the first stages of development. For example, the community health services, municipalities and provinces explored the possibility of collaborating on OPM adaptation, and the provinces organised meetings to support the development of regional coordination for OPM adaptation. In addition, some municipalities were in contact with other regional landowners (e.g., forest services and site managers). In most other cases, however, municipalities do not collaborate with other landowners on OPM adaptation. Other landowners are also not likely to collaborate with each other. Most of the interviewees identified this lack of collaboration as a barrier to effective OPM adaptation. For example, if one landowner takes adaptive measures and another does not, the OPM could spread again in an area where it had already been removed.

To summarise, the current field of actors involved in OPM adaptation is relatively complex, making the positioning of actors almost impossible. At the national level, there are OPM adaptation networks that focus primarily on research and policy advice. At the regional and local levels, early networks are evolving towards the development and sharing of knowledge. Although emerging networks provide an opportunity for improving the effectiveness of OPM adaptation, collaborative initiatives have been limited. According to a number of interviewees, the coordination of these networks could be further improved, and they expressed a need to learn from other actors in order to find effective OPM adaptation strategies. Three themes emerged from the analysis of current activities in OPM adaptation: technical, organisational and normative issues. These themes are elaborated below.

### 3.2. Technical Issues: Implementation of OPM Adaptation Strategies

Technical issues are knowledge gaps that challenge the development and implementation of OPM adaptation strategies. According to the interviewees, finding suitable OPM adaptation strategies is complicated by knowledge gaps concerning the ecological behaviour of the OPM, including techniques for controlling the OPM, the health impact of the OPM, the organisation of OPM adaptation and communication towards citizens. Although the list of knowledge gaps is too extensive to elaborate on within the context of this article, the complete list is included in Appendix A. In this section, we provide several examples for each theme, including what happens in practice because of and/or despite the knowledge gaps.

First, knowledge gaps about the ecological behaviour of the OPM, including the effects of specific control techniques on the OPM, present a challenge to the effectiveness of OPM adaptation strategies. For example, the interviewees note that, despite the application of a wide variety of adaptation strategies, the long-term environmental impact of applying organic or chemical agents and of increasing biodiversity is unclear. They also noted that some contactors offer potentially ineffective control methods (e.g., garlic concentrates and OPM traps). Some landowners lacked sufficient knowledge on control methods and contracted with people to implement such unsubstantiated and unprofessional control methods. For example, one landowner ordered several hundred OPM traps, not knowing that this method is ineffective. In addition to a lack of evidence on effective methods, interviewees from the provinces, the community health service and landowners noted that the knowledgeability of OPM control contractors is questionable. They argued that not all OPM control contractors know when to use preventive measures, even though their effectiveness depends on the timing of their application.

“So, it can be difficult. What you see is that there are a lot of cowboys offering all kinds of control methods, and that’s a problem, because all municipalities have to do something. These cowboys launch the most fantastic control methods, like garlic preparations and onion concentrates, none of which are effective.”

(Interview 8, Policymaker)

Second, the knowledge gaps concerning the ecological behaviour of the OPM and the effect of control techniques also raise questions with regard to the public health impact of the OPM and adaptation strategies. Interviewees noted that, among others, the impact of OPM adaptation strategies (e.g., increasing biodiversity and providing information about OPM to inform citizens) on public health is unclear.

“If such monitoring does not yield hard evidence of the health concern—and we have yet to be successful in this regard; the best we can say is that the OPM is annoying and itching—no one will have much interest in intervening.”

(Interview 23, Social Nurse)

Other questions concerned the organisation of OPM adaptation. There was a lack of available contactors for the application of organic and chemical agents and for the removal of nests with a vacuum. For example, one landowner described having hired a foreign contractor to remove OPM nests which challenged communication due to language barriers. Landowners further noted that there only a few professional contractors available at that time.

In addition to identifying these organisational questions, all of the interviewees mentioned informing citizens as an important measure for limiting the health impact of the OPM. It was noted that municipalities and general practitioners had received an overwhelming number of calls and complaints from citizens. This created an additional workload, thus making it difficult for municipalities to focus on effective OPM adaptation and increasing the burden of some general practitioners. Respondents from municipalities tried to address this issue by setting up an online registration tool for citizens to report OPM nests. Respondents from a municipality and community health services mentioned that the advice provided to citizens was not yet fully developed and comprehensible for all citizens. They mentioned that these problems were exacerbated by a general lack of knowledge on effective OPM adaptation strategies. It was unclear which information could be provided to citizens about appropriate methods and tools that they could apply to control OPM or to reduce their symptoms, or on how citizens could become involved in OPM adaptation. Nevertheless, most interviewees noted that citizens should be involved in OPM adaptation and that informing citizens might also increase understanding, thus possibly enhancing their willingness to adapt.

The implementation and impact of OPM adaptation strategies are thus accompanied by many issues and difficulties: a lack of available professional contractors; the presence of contractors offering ineffective control methods, variations in the knowledgeability of landowners with regard to distinguishing the effectiveness of the methods, and ambiguity concerning what and how to advise and involve citizens. The development of OPM adaptation strategies was thus complicated by a lack of evidence and other practical issues, and the impact of the OPM adaptation strategies on public health was unclear. In addition to the technical and practical implementation of OPM adaptation, the management of effective OPM control requires the organisation of OPM adaptation. This aspect is described in the following section.

### 3.3. Organisational Issues of OPM Adaptation

Organisational issues are issues that arise when actors organise OPM adaptation. They include preparation, insight into the division of tasks, expertise and coordination for OPM and the budget for OPM control.

The explosive spread of the OPM came as a surprise to most landowners, policymakers and contractors. Most actors were therefore not prepared and were searching for effective OPM adaptation strategies.For example, in the South, the actors have been dealing with the OPM for over 25 years. Since that time, municipalities, contractors and community health services have experimented with adaptation methods and investigated the health impact of the OPM on humans and animals. During periods without major outbreaks, however, OPM experiments and adaptation activities were put on a back burner. It was noted that national and regional willingness to act tends to increase when there is an outbreak, but it tends to subside when the issue is no longer urgent. This short-term, outbreak-oriented approach was of a highly ad hoc nature, and it failed to produce any sustainable, long-term OPM adaptation strategies. Although four interviewees (a physician and medical-environmental specialist; a municipal policymaker; a provincial greenspace project manager; and a site manager at a knowledge institution) did expect an outbreak of the OPM, none of them was prepared for the intensity of the outbreak once it actually occurred. This was due to a low sense of urgency at the organisations with which they were affiliated, which had led to a limited amount of financial (or other) support. In addition, some of these actors had issued warnings about OPM outbreaks as far as the ministerial level. According to one interviewee, the overall sense of urgency at the level of policy and administration was, as so little was known about the timing and possible impact of an outbreak, and adaptation activities were thus limited. The interviewees from the ministry noted that OPM adaptation is the responsibility of municipalities and landowners. For this reason, there was no national OPM adaptation strategy, and the response to the OPM was largely *ad hoc*. 

The interviews highlighted a lack of clarity within and between organisations concerning what needed to be done and by whom. For example, a sports organisation renting a sports field noted that they had no agreements about OPM adaptation with the landowner, which resulted in ineffective OPM adaptation and complaints by affected citizens. One respondent from a province noted that OPM adaptation was assigned to the road-safety department, and OPM adaptation strategies were developed without any input from ecologists or health professionals. Other interviewees noted that OPM adaptation was regarded as a problem for landowners. Landowners thus played a major role in OPM adaptation, even though they are not perceived as experts on public health.

“Everyone says that it’s a problem for landowners. But landowners aren’t knowledgeable about the health aspects, the areas that call for attention or the [public-health] risk factors. There are other organisations with a lot more knowledge about these matters. That’s another advantage of addressing the problem together.”

(Interview 9, Policy advisor)

A respondent from a community health service indicated that landowners assume too much expertise on the part of contractors and that these contractors lack the critical view that is needed in order to ensure more efficient OPM control. At the same time, however, landowners noted that they could not be too critical with regard to the level of professionalism, given the limited availability of contractors. They further noted that the lack of contractors and the high demand for OPM control allowed contractors to charge higher prices for their services. Some interviewees argued that these market conditions had contributed to the ineffectiveness of OPM control. One of the contractors noted that the organisation had been reluctant to let their own arborists remove OPM nests, as they were afraid that their employees would become allergic to the OPM, such that they would not be able to continue their jobs. They therefore hired external contractors to remove the OPM nests.

“It’s also important that any contractors you might bring in will also have the opportunity to carry out their work—that the situation doesn’t devolve into a fight or price war over who will get the contractor. […] We had already engaged a contractor who found that he could earn a little more elsewhere. He took that job.”

(Interview 15, Team manager)

It was also unclear whether any specific individual or organisation would assume a coordinating role. As described earlier in this article, some collaborative networks were established. Most of the interviewees nevertheless noted a lack of coordination in the approach to the OPM and indicated that a more integrated approach would benefit OPM adaptation. Some interviewees expected that actors would not feel any sense of urgency to establish collaborative OPM adaptation strategies unless they are certain of the health impact of the OPM.

Respondents from municipalities, provinces, executors of OPM control and landowners identified limited funding as an obstacle to OPM adaptation. None of the interviewees had a designated budget for OPM adaptation. Funding used for OPM control was part of the funding that was reserved for pest control, unforeseen circumstances or general subsidies for nature maintenance.

“I think that it will also be a financial challenge. I would obviously prefer to hire 10 groups of contractors to vacuum the OPM, but we don’t have the financial means to keep up with 90,000 trees.”

(Interview 2, Advisor public green)

Limited preparations, ambiguity about tasks and expertise, lack of coordination and limited budgets for OPM adaptation thus led to ad hoc OPM adaptation strategies. Interviewees noted a lack of coordination and regarded the current way of applying stand-alone OPM adaptation strategies as ineffective. The resources for effective OPM adaptation was thus limited. The availability of resources, including financial means, were related to the range of values that influence decision-making on OPM adaptation strategies. We discuss these values in the following section.

### 3.4. Normative Issues: Values for Decision-Making on OPM Adaptation

**Figure 2:**
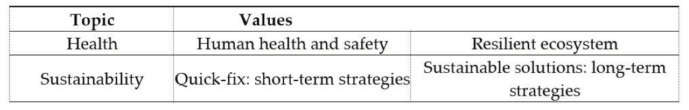
Framework of values framework for decision-making on OPM adaptation, as emerging from the interviews.

Normative issues include the range of values held by actors in the decision-making process surrounding OPM adaptation. The interviewees mentioned a variety of sometimes conflicting values for decision-making on OPM adaptation (Figure 2). The following values were mentioned: (1) health: human health and safety, and a resilient ecosystem; (2) sustaianbility: quick-fix, short-term strategies and sustainable solutions. In addition, these values are also related to the perception of risks and the division of values can be summarised under the headings of health, sustainability, risks and responsibilities. In many cases, these norms and values influence decision-making processes in which actors must choose between values, and in which trade-offs must sometimes be made. Trade-offs occur either ‘within’ actors who must make decisions or ‘between’ actors who are collaborating (or seeking to collaborate). Although these values are held separately, they are interconnected. The values mentioned by the interviewees are described below. An elaborated description of norms and values for decision-making on OPM adaptation, as emerging from the interviews, is included in Appendix B.

The first, overarching factor is health, which entails human health and safety, long with a resilient ecosystem. It was noted that decreasing the health risks will require quick adaptation strategies within the living environment. The interviewees also mentioned, however, that nature is out of balance and that it will be able to recover by itself in time. For this reason, increasing biodiversity might offer a solution to the OPM problem.

“So, we have deliberately chosen to focus on health; we do not want any health complaints […] What we actually want to do, however, is to restore biodiversity, so that the application of exterminating agents will ultimately be unnecessary. The fact of the matter is, when we apply these agents, we are intervening in the ecosystem, and that always has an effect.”

(Interview 9, Policy advisor)

This observation is related to the second factor: sustainability. In addition to quick-fix, short-term strategies (e.g., applying pesticides), there are strategies of a more long-term nature (e.g., increasing biodiversity). The interviewees noted that short-term measures can be needed to ensure health and safety for people. Although this can be done by using pesticides, it is known that pesticide also have an impact insects other than the OPM, which is harmful to biodiversity. The interviewees also mentioned that increasing biodiversity might offer a long-term sustainable solution for reducing the OPM population, although such a transition would necessarily include a period in which the OPM will have an impact on public health.

These values are related to how different actors deal with risks. In some places, pesticides are widely used, even though the long-term impact of pesticide use on the ecosystem is unknown. For this reason, several interviewees expressed reluctance to use pesticides. A representative from a nature association mentioned that preserving nature and increasing biodiversity is a great strategy, although the effect of biodiversity on the OPM remains unclear. It was also argued that humans have made the climate suitable for the OPM, and that it would be contradictory for OPM adaptation strategies to cause even more damage to nature.

“We aim to solve this problem by increasing biodiversity […] A few years are needed in order to ensure that biodiversity is in order, however, and health complaints will occur during this interim period. And this raises questions concerning whether people and municipalities will be willing to say, ‘We are not doing anything for a few years, because we want to increase biodiversity; we want to work towards this method of adaptation’. This is an area of tension. It’s difficult.”

(Interview 8, Policymaker)

The risks also include threats to human health. While some interviewees noted the need to avoid health risks, others mentioned that the exact impact of the OPM on the health of citizens is unclear. In addition, some interviewees argued that citizens could also decrease the health risks themselves, and that there needs to be some level of willingness to accept health risks to humans.

“The reason we take OPM control measures is to ensure Public Health. People live here and we have lots of people walking around, cycling and doing sports here. You can’t just let the OPM be because people suffer a lot from it.”

(Interview 12, Site manager)

“What we don’t want is an increase in severe cases. Prevention should therefore focus on control, and that is very difficult. If you ask me whether the OPM a very big problem right now, I might be the first to say, ‘Not at all, not in terms of health’. At the same time, however, I know people who have gone into anaphylactic shock due to the OPM, but that also happens when they are stung by wasps or other insects. […]. Nature has been disturbed, and we have to fix that, but we shouldn’t address it only at the back end by treating the people.”

(Interview 3, Physician and medical-environmental specialist)

Last, the values and dealing with risks also show that different actors have different responsibilities. As described by participants in the interviews, both tree owners and citizens have responsibilities with regard to OPM adaptation. All of the interviewees mentioned that tree owners have a legal duty to ensure safety around their trees (although different tree owners interpret and implement these duties differently). Some interviewees, however, argued that citizens themselves should take more responsibility (e.g., by wearing protective clothing). They mentioned that citizens should accept that the OPM is part of the ecosystem and that people should take this into account and adapt their behaviour during certain times of the year.

As reflected in the topics of health, sustainability, risks and responsibilities, different actors have different reasons for deciding on specific OPM adaptation strategies. For example, some actors focus on public health, while others focus more on a resilient ecosystem. Such contrasts do not necessarily require trade-offs. For example, a landowner can decide to use pesticides in busy places (e.g., nearby schools or car parks) in order to ensure the health and safety of humans, while also taking measures to increase biodiversity. At the same time, however, there is a risk that citizens will be affected by OPM setae, and there is a risk that pesticides will damage the ecosystem. There is thus no single ‘right’ strategy; decisions on OPM adaptation are influenced by a range of values. As demonstrated by these results, it is important to take values into account when developing strategies for OPM adaptation. The development of OPM adaptation strategies involves more than simply arriving at technical solutions, as the choice of technical strategies to be used is necessarily determined by underlying values.

## 4. Discussion

In response to the limited understanding concerning PHA and the challenges that professionals face with regard to the development of PHA strategies, this study focuses on a specific PHA issue that is exacerbated by climate change: adaptation to the oak processionary moth [24]. This study intended to explore and describe the dilemmas and complexities involved OPM adaptation by mapping actors, networks, activities and challenges involved in OPM adaptation.

This exploration showed that there is a complex network of interactions regarding OPM control. There is a range of different public and private landowners. In addition, other actors are also involved with the OPM, such as citizens, contractors, waste operators, actors involved in research, advise, policy and administration and health professionals. At the national level, actors such as ministries and the interprovincial counsel influence OPM adaptation. Therefore, the development of PHA strategies for OPM control involves policies of different sectors and actors with various professional backgrounds. Consequently, the actors focus on different themes which can be categorised in the themes: technical, organisational and normative issues.

In summary, the analysis of the interviews has yielded three key insights concerning how OPM adaptation is developed. First, the interviews revealed a fragmented actor field, in which early networks are emerging, but cross-sector coordination is still limited with regard to OPM adaptation. Different actors hold different values and priorities with regard to health, sustainability, risks and responsibilities. These diverse values and priorities pose a challenge to connections with other actors and, in some cases, they can act as obstacles to collaboration and the development of a coordinated approach. Second, the technical aspects of OPM adaptation indicate that adaptation strategies are not yet sufficiently evidence-based. The impact of the various OPM adaptation strategies on the OPM and on public health is unclear, and uncertainties exist with regard to the ecological behaviour of the OPM. Both scientific and practical experiential knowledge is relevant to the development of effective strategies for OPM adaptation. The generation of knowledge and its translation into practical strategies requires interdisciplinary cooperation aimed at knowledge development concerning adaptation. Finally, the division of responsibilities is unclear. Either actors do not apply any OPM adaptation strategies, or they apply OPM adaptation strategies that have been neither discussed nor aligned to the strategies of neighbouring landowners. The lack of interaction and coordination are preventing the development of an adequately integrated approach.

Our findings are important in light of the ongoing challenges associated with coping with climate change and its health effects, as well as with improving PHA strategies [1,45]. As suggested in a recent review by Anenberg S.C, Haines S [46], synergistic effects between heat exposure and air pollution driven by climate change imply that the impact of climate change on health might be greater than previously estimated. These increased health risks highlight the importance of PHA research. Previous studies have suggested that, although national commitments to PHA have increased, they are still poorly developed, due to limited government coordination, political engagement and funding [1,46]. PHA issues, such as OPM adaptation, might benefit from climate programmes and deals such as the European Green Deal in which restoring biodiversity is a major action [47]. Furthermore, the current COVID-19 pandemic has amplified the interconnected relationships between environment, climate change and public health, thereby increasing the need for PHA research [9,11]. Although the OPM issue might be considered of relatively minor importance or urgency, it nevertheless continues to pose a challenge with regard to the development of a clear and effective PHA strategy. Insight into the development of PHA strategies relating to this issue could thus enhance understanding of potentially life-threatening PHA issues, including heat stress and outbreaks of vector-borne diseases, such as the West Nile virus (WNV) [48]. Examples of WNV PHA strategies are increased surveillance, early-warning detection methods and coordinated effort among health organisations and mosquito abatement organisations to organize vector control and to reduce reintroductions and limit WNV transmission [49,50]. In addition, Rund, Moise [51] suggest using an open-access repository of mosquito surveillance data to generate data to understand vector population dynamics and collect current and future surveillance data for such databases and systems. However, in the USA and in Europe, WNV control is mainly based on local interventions. Data are unused and coordinated efforts are lacking [49,50,51,52].

In line with previous studies, our results demonstrate the complexity of decision-making processes for landowners and other actors involved with issues relating to the OPM [29,30,32]. Actors must make decisions that involve norms and values concerning health, sustainability, risks and responsibilities [29]. The process of OPM adaptation thus involves more than technical solutions alone. Other aspects include the need to develop a shared perception of and solution space for the problem and to inform citizens about the OPM adaptation strategies that they can apply on their own.The role of citizens in OPM adaptation strategies is limited. In line with other studies on adaptation, citizen involvement is often limited to the provision of information, rather than engagement in the development of PHA strategies [53,54]. As reported in a study on OPM monitoring, there is considerable enthusiasm amongst citizens for active participation in various efforts, including environmental monitoring through citizen-science initiatives [55]. In addition, the active involvement of citizens in PHA through their role as consumers and members of civil society could increase the legitimacy and awareness of adaptation, while enhancing the mainstreaming of adaptation into other activities [56,57]. Furthermore, as demonstrated by Semenza, Ploubidis [58], the motivation of citizens to adapt their behaviour to climate risks depends on their perceived vulnerability to threats, the severity of climate change, the availability of information, and the framing of climate change information. If climate change is framed from a health perspective, citizens may thus be motivated to engage in adaptive activities [58].

### 4.1. Strengths and Limitations

For this study, we interviewed a wide range of actors involved in OPM adaptation at the national, regional and local level. Some of these actors were directly involved in the development of OPM adaptation strategies. Others were involved with the OPM, but they were not (or not yet) developing OPM adaptation strategies. This variety of participants is one strength of this study, as it yielded a comprehensive overview of how OPM adaptation currently takes place. At the same time, however, we must acknowledge that the method of purposive sampling and snowball sampling used to select the interviewees could potentially be a source of selection bias. The interviewees who were selected through purposive sampling may have had more experience with the development of OPM adaptation approaches than was the case for other actors in the field. Moreover, the snowball sampling might have resulted in the inclusion of relatively like-minded interviewees. To overcome this limitation, we included actors from a variety of sectors as much as possible, in addition to including national-level actors, in order to examine whether the regional and local OPM adaptation approaches were consistent with the development of OPM adaptation at the national level. By applying qualitative research methods, we aimed to gain a deeper understanding of the development of OPM adaptation strategies and the complexities involved in OPM adaptation.

### 4.2. Future Research

We did not identify the specific OPM strategies that are applied more frequently than others, nor were we able to compare the effects of different OPM strategies. To answer these types of questions, we recommend that future studies should also apply quantitative methods. As demonstrated by the results of this study, OPM adaptation should be regarded within the social and ecological context. The various issues raised by the respondents correspond to the conceptual framework for long-term investigations of social-ecological systems, as developed by Redman, Grove [59]. This multiscale framework connects human and ecological systems, regarding them as a single, complex and integrated social-ecological system. The ecological patterns and processes interact with social patterns and processes within a push-and-pull relationship. Processes within these systems are also influenced by external political and economic conditions, as well as by external bio-geophysical conditions. Core concepts in the application of this framework include societal and scientific concerns relating to processes of change; comparison amongst sites; the ambition to explain and develop prognoses; and theory-building efforts [59,60].

Further research could focus on social learning behaviour by examining reciprocal interaction between personal, behavioural and environmental influences (e.g., through the application of social learning theory) [61,62,63]. Particularly given that the OPM has been present in The Netherlands for 25 years and adaptation efforts to date have failed to result in any sustainable, long-term adaptation approach, social learning could support the efforts of responsible organisations, contractors and the public to develop an understanding of PHA strategies, while enhancing a process through which mutual understanding can develop from the interaction between science and practical experience [22,64]. The potential of social learning within complex social-ecological challenges (e.g., PHA) could clarify implications for governance and other interventions that call upon actors to find adequate adaptation strategies [65]. Participatory action research methods, such as reflexive monitoring, can help analyse and facilitate social learning processes among the diverse sets of actors [66,67,68].

## 5. Conclusions

The findings of this study indicate that OPM is an emerging PHA issue that involves a wide range of actors who are fundamentally dependent on each other for the development and execution of effective OPM adaptation strategies. At the same time, however, different actors have different norms, values and priorities with regard to health, sustainability, risks and responsibilities, and this poses a challenge to collaboration. In addition, current OPM adaptation strategies are insufficiently substantiated or evidence-based, and the impact of most of these strategies on the health of humans and the ecosystem remains unclear. As actors are confronted with ambiguities and uncertainties concerning OPM adaptation, there is no single acceptable solution for addressing the problems associated with the OPM. Better interaction and coordination are therefore needed, in order to develop the knowledge needed to arrive at an adequate, integrated approach. The generation of knowledge and its translation into practical strategies therefore calls for interdisciplinary cooperation in knowledge development. The current COVID-19 pandemic has amplified the interaction existing between the environment, climate change and public health. Emerging PHA issues (e.g., the West Nile virus) require the development of PHA strategies. As demonstrated by this study, adaptation involves more than technical solutions alone. Other relevant aspects include the development of a shared perception of and solution space for the problem, and the active engagement and participation of citizens in OPM adaptation enabling them to apply adaptation strategies as well. Social learning theory and methods, such as reflexive monitoring, could clarify the implications of these strategies for governance and other interventions that call upon actors to find adequate adaptation strategies.

## Figures and Tables

**Table 1:** OPM adaptation strategies.

Focus of Strategy	Strategy	Actors Responsible
Ecology		
	OPM control: Natural control measures (e.g., increasing biodiversity) Application of organic or chemical agents Curative control (e.g., fixating, isolating and removing nests and setae) Planning and prioritising the application of insecticides or pesticides (including bio-pesticides) and nest removal	Landowners Contactors (commissioned by tree owners) Community health services
Humans		
	Providing information about OPM to inform citizens	Community health services Municipalities
	Avoiding affected oak trees and contact with grass, benches, water and other objects affected with setae	Citizens
	Treatment of mild symptoms	General practitioners
	Use of ointments	Citizens
	Symptomatic treatment Management of complications	Specialist service Hospital care

**Table 2:** Interview respondents.

Respondent	*n*	Organisation	Occupation or Function	Region
1	1	General practice	General practitioner	East
2	1	Municipality	Advisor public green	South
3	1	Health and environment consultancy	Physician and medical environmental specialist	South
4	1	Community health services	Physician and medical environmental specialist	South
5	1	Regional site-management organisation	Forester	South
6	1	-	Private tree owner	South
7	2	Nature association	Coordinator and secretary	South
8	1	Province	Policymaker	East
9	1	Municipality	Policy advisor	East
10	1	Province	Green space project manager	South
11	1	Ministry	Policy advisor	National
12	1	Knowledge institution	Site manager	East
13	1	Municipality	Public-space manager	South
14	1	Nature association	Ecologist	National
15	1	Waste operations company	Team manager	East
16	1	Sports association	Member of sports association	South
17	2	Regional site-management organisation	Team manager and forester	East
18	1	Sports association	Team manager	East
19	1	Municipality	District manager	East
20	1	Regional site-management organisation	Ecologist	East
21	1	Ministry	Policy advisor	National
22	1	Maintenance contractor	Technical manager	South
23	1	Community health service	Social nurse	East
24	1	Regional site-management organisation	Ecologist	East

## Data Availability

Not Applicable.

## Appendix A

**Table A1:** OPM Knowledge Gaps.

Theme	Questions
**Communication**	- Which form of public information about OPM is most effective? ∘ What is the best way to inform citizens about OPM, and which media can be used for communication? For example: ▪ What is the best way to inform citizens about swimming water contaminated by OPM setae? ▪ What is the best way to inform citizens be informed about the long-term impact of OPM and the long-lasting effects of the setae? - What advice can be given to citizens (e.g., by community health services, municipalities, landowners) about OPM adaptation, and what action perspective can be offered? ∘ What information can be provided to citizens about self-protection and self-treatment? For example, ▪ Is it advisable to recommend that citizens should to go to a sauna or use a blow-dryer if they experience itchiness due to the OPM? ▪ Is it advisable to recommend that citizens should keep windows closed to prevent OPM setae from entering, and what consequences does this have for ventilation?
**Health**	- What is the severity of the health complaints caused by OPM? ∘ How severe must a health complaint caused by the OPM be for it to constitute a health problem? ∘ How many people develop serious health complaints (e.g., allergies or anaphylactic shock) due to OPM? - Could repeated exposure to OPM contribute to increased sensitivity and/or allergy to OPM? - What is the effect of adaptation on health complaints (and their reduction)? - When should people with OPM complaints be advised to consult with their physicians? - What is the best way for citizens and professionals who spend time in the forests to protect themselves? - What do citizens do when they experience OPM health problems? ∘ Are citizens aware of measures they can take to prevent and/or reduce OPM health complaints? ∘ What can be expected of citizens with regard to the prevention of OPM complaints?
**Ecology and control**	- What is the long-term impact of the current practice of applying organic or chemical agents? - What is the impact of increasing biodiversity on the OPM? - What is the relationship between OPM and the presence of nitrogen deposition? - How effective are caterpillar traps? - How long do OPM stay in the ground during a diapause, and to what extent can this serve to extend the OPM season? - Exactly how do pheromone traps provide an indication of the expected number of OPM in the next season? - Which alternative methods are suitable for controlling the OPM? (e.g., foil, garlic)? - What is the impact of OPM nests that remain in trees on biodiversity and the control of OPM? - How can OPM setae be neutralised? - At which temperature should clothes be washed in order to neutralise OPM setae?
**Governance and organisation**	- Which applications are allowed for use in controlling OPM? - Which parties are best suited to coordinate OPM adaptation? - How can contractors be encouraged to increase their knowledge and skills with regard to OPM adaptation? - How can cooperation between site managers be promoted in order to achieve a more effective approach to OPM adaptation? - What is the most cost-effective form of OPM adaptation?

## Appendix B

**Table A2:** Elaborated Description of Norms and Values for Decision-Making on OPM Adaptation, as Emerging from the Interviews.

Actor Group	Norms and Values
Policy and administration & land owners (I.2,8,9,10,13,19)	- Enhancing biodiversity and restoring nature in the long term, including increasing the presence of natural predators - Ensuring safety around trees - Health and safety of residents in a healthy environment - A more green and sustainable climate in the city - Informing citizens on nature (conservation) - Road safety
Land owners (I.5,6,12,17,18,20,24)	- Ensure health and safety of people - Maintenance of parks and lanes - Increasing biodiversity restoring natural balance, and nature conservation - Healthy ecosystem - Ensure safety and prevent human contact with the OPM - Inform visitors about OPM
Policy and administration (not land owners) (I.11,21)	- Safety for people in nature - Reducing invasive species and plagues
Community health service (I. 3,4,23)	- Preventing public health risks - Preventing overburden of the healthcare system - Provide information to citizens about OPM - Clean and safe living environment - Restoring the ecosystem
Implementation of OPM control (I.22,15)	- Control pests and diseases (including OPM) - Reassuring citizens that it is safe outside - Take targeted preventive measures against EPR - Do not harm vulnerable species - Increasing biodiversity
Representatives (I.7,14)	- Preserving nature and increasing biodiversity - Inform actors about the effect of the ecology of the OPM and pesticides - Raising awareness among citizens about the preventive measures
